# The emerging role of vascular endothelial cell-mediated angiogenesis in the imbalance of RA synovial microenvironment and its clinical relevance

**DOI:** 10.3389/fphar.2025.1481089

**Published:** 2025-04-04

**Authors:** Xingxing Huo, Yanhui Peng, Hui Li, Chen Li, Faxue Liao, Chenggui Miao, Yurong Huang

**Affiliations:** ^1^ Experimental Center of Clinical Research, The First Affiliated Hospital of Anhui University of Chinese Medicine, Hefei, China; ^2^ Department of Pharmacology, School of Integrated Chinese and Western Medicine, Anhui University of Chinese Medicine, Hefei, China; ^3^ Department of Orthopaedics, The First Affiliated Hospital, Anhui Medical University, Hefei, China; ^4^ Center for Xin’an Medicine and Modernization of Traditional Chinese Medicine of IHM, Anhui University of Chinese Medicine, Hefei, China; ^5^ School of Chinese Medicine, Li Ka Shing Faculty of Medicine, The University of Hong Kong, Pokfulam, China; ^6^ Department of Respiratory Medicine and Center of Infection and Immunity, The First Hospital of Jilin University, Changchun, China

**Keywords:** rheumatoid arthritis, vascular endothelial cells, angiogenesis, fibroblastoid synovial cells, macrophages, post-translational modification

## Abstract

Vascular endothelial cells (VEC) play a key role in the occurrence and progression of vascular inflammation. VEC activation secretes powerful inflammatory mediators and aggravates the development of rheumatoid arthritis (RA). Angiogenesis plays a key role in the pathological processes of inflammation and synovial infiltration, driving RA progression. A substantial amount of evidence suggests that the VEC at the inflammatory site of RA is both an active participant and a regulator of the inflammatory process. At present, the research progress of VEC and inflammation in RA is still incomplete. In this review, we summarize the role of VEC and angiogenesis in the development of RA, describe the relevant cells, cytokines and signaling pathways involved in regulation, and provide research clues on the role of post-translational modification (PTMs) in VEC function and angiogenesis in RA, and classify and integrate these mechanisms and therapeutic strategies. This review aims to synthesize current evidence to support the established link between VEC and RA-related pathology, provide a theoretical basis for clinical studies, and provide valuable insights into the development of therapeutic drugs against RA.

## Graphical Abstract

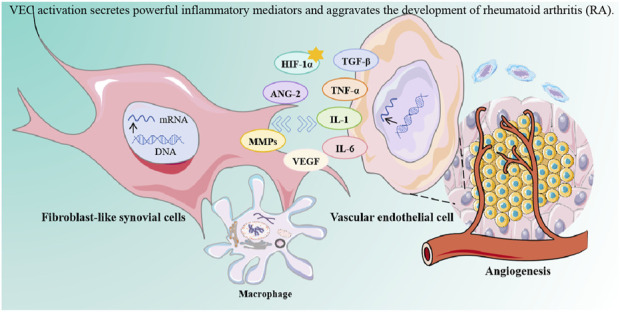

## 1 Introduction

Rheumatoid arthritis (RA) is a chronic systemic autoimmune disease, and the incidence of RA increases with age (Luo et al., 2023). Multiple genetic factors and environmental risks are closely related to the development of RA. The pathological features of RA include chronic inflammation of the synovium and destruction of bone and cartilage (Smolen et al., 2016). Central to RA-related bone loss is the imbalance between osteoclast-mediated resorption and osteoblast-mediated repair (Komatsu, 2022). In the progression of RA, due to the rapid proliferation and activation of inflammatory cells such as vascular endothelial cells (VEC), fibroblast-like synovial cells (FLS), T cells, B cells and macrophages, abnormal synovial tissue expansion, angiogenesis and intimal hyperplasia occur. A large number of inflammatory cytokines such as tumor necrosis factor (TNF-α), interleukin-1β (IL-1β), and prostaglandins, as well as aggressive proteases such as matrix metalloproteinases (MMPs), are infiltrated, leading to injury of cartilage and bone tissue (Cho et al., 2007).

Vascular endothelial cells (VECs), positioned at the blood-tissue interface, play dual roles in RA: they regulate immune responses and vascular homeostasis but also contribute to pathological angiogenesis and inflammation. Notably, synovial vascular endothelial growth factor (VEGF) concentration was significantly higher in male than in female RA patients. Despite this, the responsiveness of vascular endothelium to SF priming was higher in females, suggesting that gender differences in angiogenic responses were mainly related to the endothelial genotype, sex-specific differences in VEC function—potentially linked to hormonal influences—may partially explain the higher RA prevalence in women (Baggio et al., 2022; Clapp et al., 2016). Under inflammatory conditions, activated VECs secrete mediators that recruit immune cells (e.g., T/B lymphocytes, macrophages) and perpetuate synovial hyperplasia, forming invasive pannus tissue (Bloom et al., 2023; Zimmermann-Geller et al., 2019). This tumor-like synovial proliferation, fueled by aberrant angiogenesis, accelerates cartilage and bone destruction through direct osteoclast activation and protease release (Jang et al., 2022; Ortiz et al., 2020).

Angiogenesis is an early and critical event in the pathogenesis of RA, and this process is regulated by angiogenesis stimulating factors and angiogenesis inhibitors (Gao et al., 2024). Normally tightly regulated, vascular growth becomes pathological in RA due to imbalances between pro-angiogenic factors (e.g., VEGF) and inhibitors (Dudley and Griffioen, 2023). Newly formed vessels not only supply nutrients to hyperplastic synovium but also recruit inflammatory cells, creating a self-perpetuating cycle of joint damage (Wang et al., 2021a).

**FIGURE 1:**
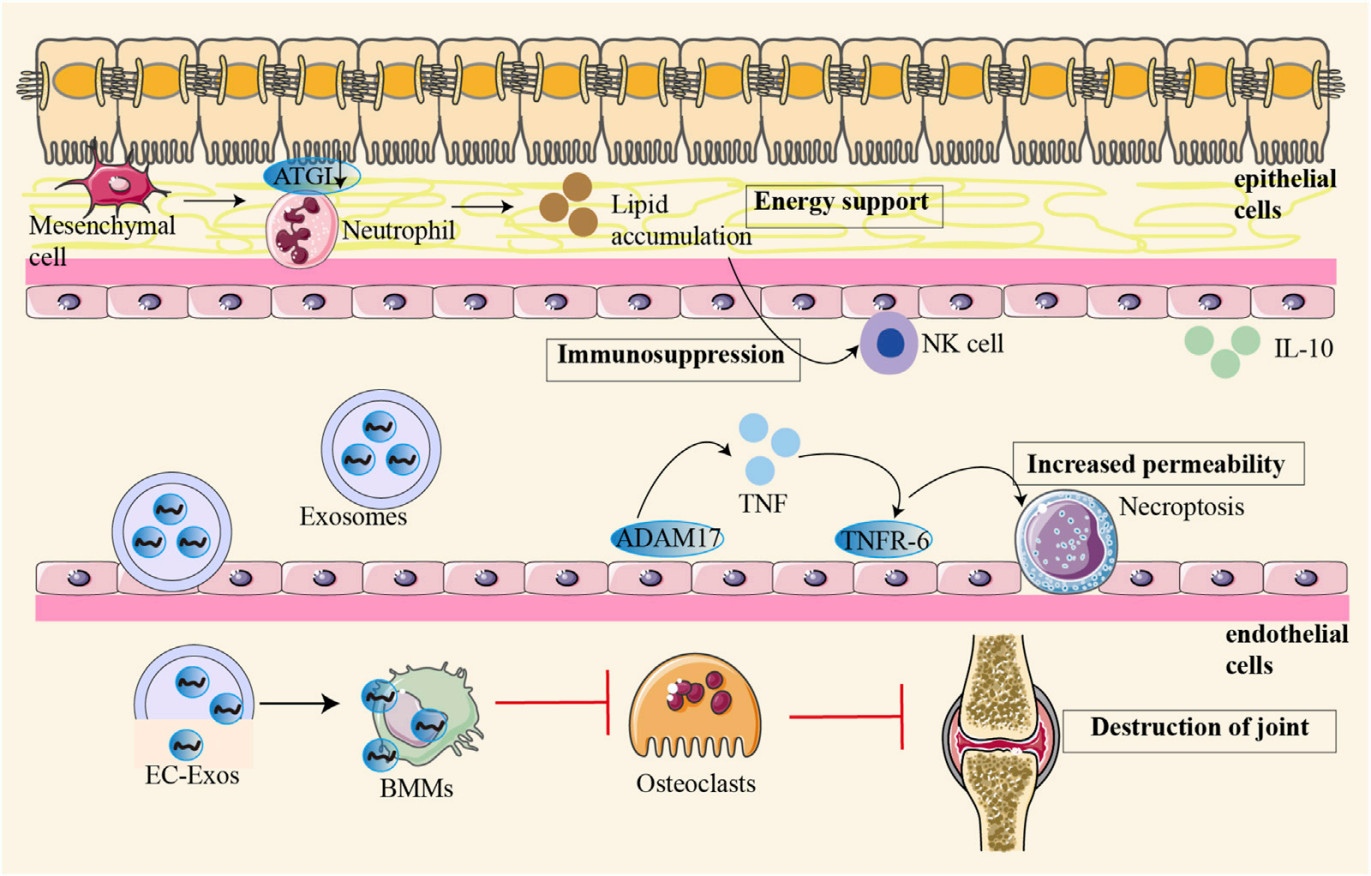
Osteoblast-derived VEGF regulates osteoblast differentiation and bone formation during bone repair. The pannus in the synovium of RA showed tumour-like hyperplasia, and this abnormal angiogenesis led to cartilage degradation and bone destruction. In pathological conditions, different cells interact with each other to aggravate joint destruction. Endothelium-derived exosomes interact with bone marrow-derived cells to inhibit osteoclast formation and ultimately lead to joint destruction. Angiogenesis may directly stimulate osteoclast precursors and osteoclasts, further aggravating joint destruction.

Tumor angiogenesis is dynamically regulated by infiltrating bone marrow cells such as macrophages, myelo-derived suppressor cells (MDSC), and neutrophils (Yang et al., 2024). The pannus in the synovium of RA showed tumour-like hyperplasia, and this abnormal angiogenesis led to cartilage degradation and bone destruction. Endothelium-derived exosomes interact with bone marrow-derived cells to inhibit osteoclast formation and ultimately lead to joint destruction. In addition, angiogenesis may directly stimulate osteoclast precursors and osteoclasts, further aggravating joint destruction (Figure 1) (Hu and Olsen, 2016). These pathological processes not only cause damage to the joint structure, but also increase the number of local pain receptors, thus causing pain. In addition, the invasion of inflammatory cells and angiogenesis promote each other, forming a vicious cycle that exacerbates inflammation and destruction of the joint. Therefore, therapeutic strategies targeting angiogenesis play an important role in the treatment of RA (Elshabrawy et al., 2015).

Recent studies suggest a strong association between VECs and RA-related pathology, highlighting their potential role in disease progression.This review synthesizes mechanisms by which VECs and angiogenesis drive RA progression, explores sex-specific knowledge gaps, and evaluates emerging therapeutic strategies targeting these pathways.

## 2 VEC mediates angiogenesis and participates in RA pathology

Angiogenesis in RA synovium is a tightly regulated process orchestrated by coordinated interactions between vascular endothelial cells (VECs), vascular smooth muscle cells (VSMCs), pericytes, and immune cells through interconnected signaling networks (Jiang Y. et al., 2023). At present, it is believed that the specific mechanism of angiogenesis in RA synovial tissue is that the angiogenic medium activates VEC through protease. These proteases degrade the basement membrane to make VEC grow outward to form capillary buds. VEC mitosis in the bud, other cells at the tip of the bud migrate but do not proliferate, forming a cavity. The buds anastomosed with each other to form a capillary ring and synthesize a new basement membrane (Szekanecz et al., 2009).

**FIGURE 2:**
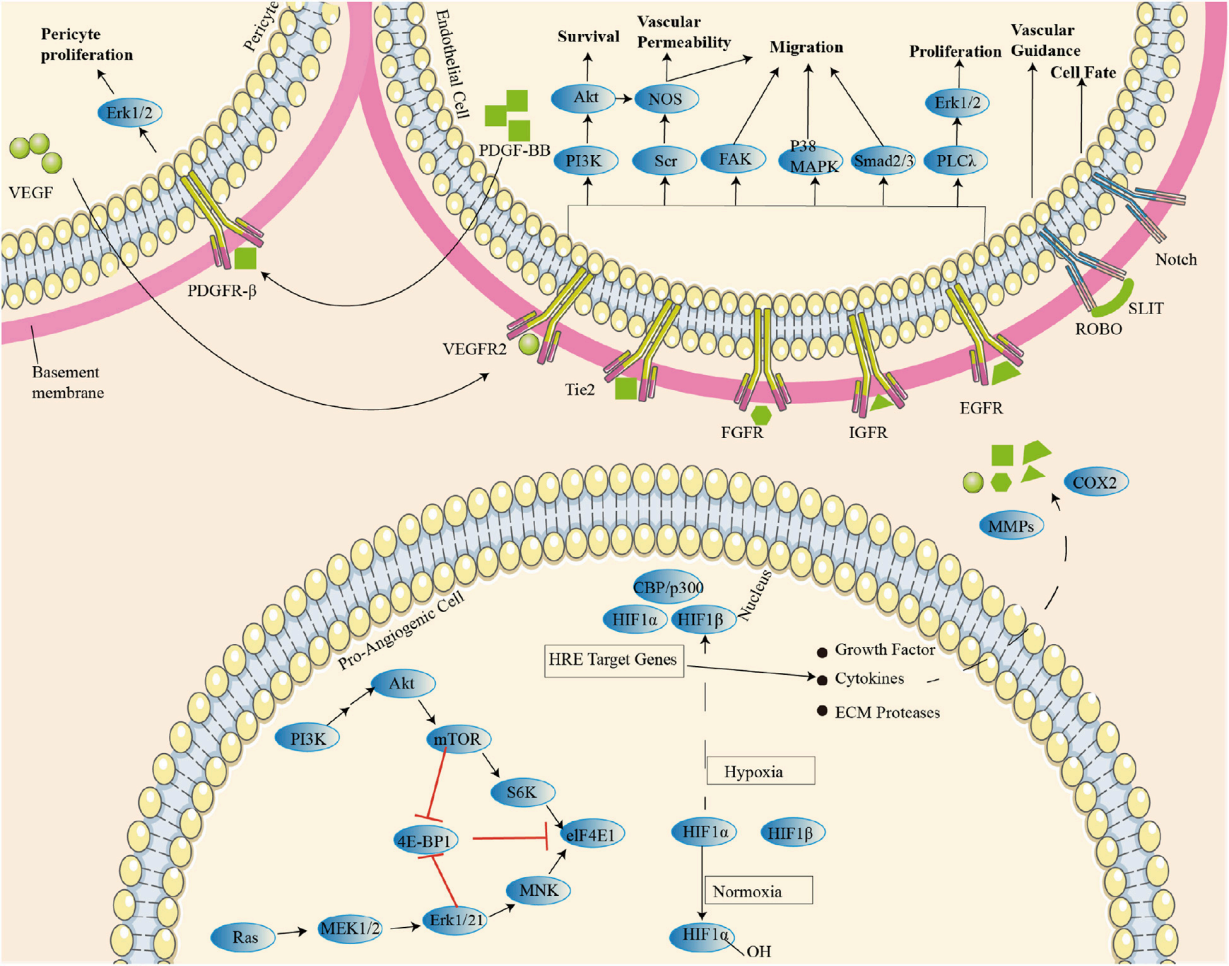
Regulatory mechanisms of various cellular and molecular signaling pathways during angiogenesis: Parietal cells bind endothelial cells (EC) by expressing ANG-1, leading to TGF-β activation and ECM sedimentation, thus stabilizing the growing vascular bed. Downstream effectors (including PI3K, Src kinase, FAK, p38 MAPK, Smad2/3, and PLCγ/Erk1/2) promotes endothelial cell survival, vascular permeability, and migratory/proliferative phenotypes.

With vascular maturation and hemodynamic changes, VEC secretes platelet-derived growth factor (PDGF-B) to recruit pericardial cells and VSMC. These parietal cells bind to VEC by expressing angiopoietin 1(ANG1), leading to TGF-β activation and extracellular matrix (ECM) sedimentation, which stabilize the growing vascular bed. Downstream effectors, including phosphatidylinositol-3 kinase (PI3K), Src kinase, adhesion spot kinase (FAK), p38 mitogen-activated protein kinase (p38 MAPK), Smad2/3, and phospholipase Cγ (PLCγ)/Erk1/2, promote VEC survival, vascular permeability, and migration/proliferative phenotypes. Regulation of positive and negative transcription of these semigroups groups by microRNA (miRNA) can further affect the blood vessels after angiogenesis (Figure 2) (Adams and Alitalo, 2007; McColl et al., 2004). Exosomes, extracellular vesicles critical for intercellular communication, play a pivotal role in RA angiogenesis by shuttling bioactive molecules such as miRNAs and proteins between cells (Zhang et al., 2015). Expression of miR-200a-3p is significantly increased in TNF-α-induced exosomes and in exosomes-treated human umbilical vein endothelial cells (HUVEC), which suppresses the nuclear transcription regulator KLF6 in human umbilical vein endothelial cells (HUVECs). This inhibition elevates VEGFA expression, enhancing HUVEC migration, invasion, and angiogenesis via the miR-200a-3p/KLF6/VEGFA axis (Zhang et al., 2023). While HUVECs provide critical insights into endothelial mechanisms, limitations include their origin from umbilical veins rather than synovial vasculature and the absence of RA-specific inflammatory microenvironment cues (e.g., synovial fibroblast crosstalk, hypoxia gradients). In RA FLS, adipokine apelin (APLN) inhibits miR-525-5p synthesis through phospholipase Cγ (PLCγ) and protein kinase Cα (PKCα) signaling, thereby promoting ANG1-dependent endothelial progenitor cell (EPC) angiogenesis (Chang et al., 2021). Beyond exosomal miRNAs, epigenetic regulation by long non-coding RNAs (lncRNAs) further modulates angiogenesis. In RA FLS-stimulated HUVECs, Long non-coding RNA (LncRNA) HOTAIR is upregulated. Activation of PI3K/AKT pathway through the miR-126-3p/PIK3R2 axis, which increases VEGF, FGF2, CD34, and CD105 expression, promotes synovial angiogenesis (Liu et al., 2023). And Ezrin regulates the HiPO-Yes-associated protein one nuclear translocation and interacts with the PI3K/Akt signaling pathway, affects VEC migration and angiogenesis (Chen et al., 2021).

Synovial angiogenesis in RA consists of a cascade of multiple events, which is fundamentally due to the imbalance between the promoting mediators and inhibitors of angiogenesis. The cytokine network is complex in RA, and the angiogenesis process is caused by a variety of mediators, such as growth factors, mainly hypoxia-inducible factor (HIF) and VEGF, and various pro-inflammatory cytokines, various chemokines, matrix components, cell adhesion molecules, proteases, etc (Latacz et al., 2020). In inflammatory or hypoxic environments, HIF-1 expression is increased to mediate angiogenesis. Hypoxia stabilizes HIF-1α, which translocates to the nucleus to induce VEGF expression, linking angiogenesis to joint inflammation. VEGF then promotes the activation of VEC to induce inflammation, thereby establishing a crosstalk between angiogenesis and joint inflammation in RA (Song et al., 2023).

In the process of angiogenesis, many inflammatory cells and transmitters are produced, such as IL-1, IL-6, TNF-α, TGF-β, PG, NO, CD40L-CD40, which can upregulate VEGF expression under hypoxia. The upregulated expression of VEGF can promote the formation of persistent synovial inflammation, release a large number of inflammatory factors, aggravate joint hypoxia, and then cause an irreversible vicious cycle. For example, TNF-α and IL-1β upregulate VEGF-C in RA FLS, promoting lymphangiogenesis and angiogenesis (Cha et al., 2007). IL-17 affects RA angiogenesis by up-regulating VEGF expression in RA FLS (Ryu et al., 2006). IL-17 synergizes with TNF-α to enhance RA FLS migration and reactive oxygen species (ROS production via NADPH oxidase 4 (Lee et al., 2020). On the contrary, IL-35 can inhibit the expression of VEGF, FGF-2, TNF-α and IL-6 in FLS, and inhibit angiogenesis by affecting STAT1 signaling (Wu et al., 2018). IL-4 has an anti-angiogenic effect, especially in the inflammatory environment of RA, by inhibiting VEGF production in synovial fibroblasts. When TGF-β stimulated FLS, IL-4 showed an inhibitory effect on VEGF production. The combined treatment of IL-4 and IL-10 inhibited TGF-β-induced VEGF production in an additive manner (Hong et al., 2007). The expression levels of TLR3, VEGF and IL-8 in RA synovium are significantly higher than those in OA synovium. NF-κB inhibitors, such as pyrrolidine dithiocarbamate and parthenolide, can eliminate the stimulation of TLR3 ligand poly (I:C) on the production of VEGF and IL-8 in RA FLS (Moon et al., 2010). Therefore, targeting the TLR3 pathway may be a promising approach to prevent pathologic angiogenesis in RA.

Key regulators like CD147 and GATA4 offer therapeutic potential.The SCID mouse coimplantation model of RA (SCID-HuRAg: 6-8-week-old male NOD/SCID mice (SLAC) that had been bred under specific pathogen-free conditions were used for the experiments. A 1-cm incision was made in the left flank of each mouse. Normal human cartilage and rheumatoid synovial tissue were placed in the chamber in the muscle using fine forceps. The entire procedure was performed under sterile conditions.) (Geiler et al., 1994) was established, mice were treated with CD147 monoclonal antibody, the expression of VEGF and HIF-1α decreased more after CD147 inhibition than after infliximab treatment (Wang et al., 2012). Transcription factor GATA4 is a key regulator of cardiac differentiation-specific gene expression and is highly expressed in the synovium of RA patients. GATA4 induces angiogenesis factors VEGFA and VEGFC by directly binding to promoters and enhancing transcription (Jia et al., 2018). CD147 induces VEGF and HIF-1α in RA FLS via the PI3K/Akt pathway, while GATA4 directly binds promoters of VEGFA/VEGFC to enhance transcription.

## 3 Crosstalk between VEC and FLS in RA process

During the course of RA, FLS leads to joint damage by stimulating pro-inflammatory and tissue-destroying pathways. FLS form the joint lining, epigenetically imprinted the aggressive phenotype of RA, and play an important role in these pathological processes (Tsaltskan and Firestein, 2022). In addition to producing extracellular matrix and joint lubricants, FLS in RA also produce inflammatory mediators, such as cytokines and proteases, which are involved in the pathogenesis and continuation of RA (Nygaard and Firestein, 2020). HOXA5 is a key regulator of class 3 semaphorins expression in the synovial membrane of RA patients. TNF-α and IL-1β can decrease the expression of HOXA5 in RA FLS and HUVEC, and regulate FLS migration and invasion as well as VEC migration (Martínez-Ramos et al., 2023).

Various immune cells interact with FLS to promote and maintain local inflammation, and FLS in RA regulates the inflow of inflammatory infiltrates through crosstalk with neighboring VEC (Lei et al., 2024). The expression of cell adhesion molecules on the VEC is increased, which facilitates the capture, rolling, and stagnation of immune cells from the vasculature and the migration of immune cells into tissues (Klein, 2018). When co-cultured with VEC, FLS from inflamed joints in advanced RA patients increased the expression of adhesion molecules on VEC and promoted the adhesion of lymphocytes to VEC. Importantly, when IL-6 was added to the model, FLS from non-inflamed or fading tissues inhibited lymphocyte adhesion, while FLS from very early RA or late RA supported lymphocyte adhesion (Filer et al., 2017).

The expression of VEGF in RA joints is not only regulated by inflammatory cytokines but also by the physical interaction between activated white blood cells and FLS. The interaction of inflammatory activated white blood cells (monocytes or polymorphonuclear neutrophils(PMN)) with FLS leads to a synergistic increase in VEGF expression and secretion, stimulating VEC proliferation and endovascular formation *in vitro*. VEGF secretion levels correlate with the expression of cell surface integrins (CD11b and CD18) on monocytes and PMN in RA synovial fluid and are strongly dependent on the contact between adhesion molecules and cells (Kasama et al., 2001). Serum amyloid A (SAA) promotes the proliferation of FLS in RA. SAA activity is mediated by formylpeptide receptor-like 1 (FPRL1) receptors. SAA stimulates the proliferation, migration, and tube formation of VEC *in vitro*, and enhanced the germination activity of VEC *in vitro* and angiogenesis activity *in vivo* (Lee et al., 2006). The binding of SAA to FPRL1 may promote the destruction of bone and cartilage by promoting FLS cell proliferation and angiogenesis, thus providing a potential target for the control of.

**FIGURE 3:**
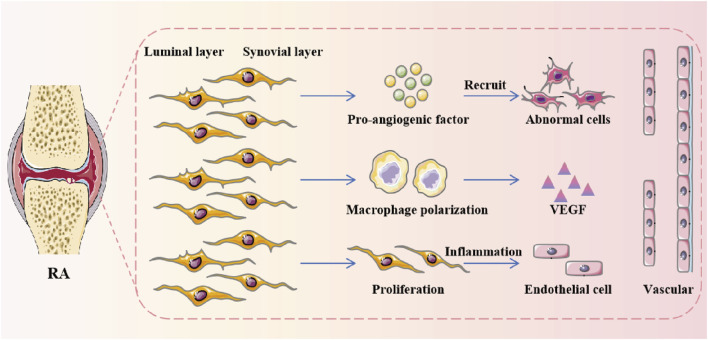
The angiogenic factors are mainly produced by RA synovial macrophages and FLS. During the formation of pannus in RA synovium, FLS promoted the polarization of resident macrophages and accelerated the secretion of pro-inflammatory mediators and pro-angiogenic factors.

The angiogenic factors are mainly produced by RA synovial macrophages and FLS (Wei et al., 2020). In the process of pannus formation in RA synovial tissue, FLS promotes the polarization of resident macrophages and accelerates the secretion of pro-inflammatory mediators and pro-angiogenic factors. The abnormal proliferation of FLS amplifies the ECS response by activating inflammation. VEC provides the pro-inflammatory phenotype of FLS to promote the development of synovitis (Figure 3) (Zhao et al., 2023). Notch signaling pathway is involved in the stabilization and angiogenesis of VEC differentiation. JAG1, DLL4, and Notch1 are highly enriched in RA ST lining and sublining CD68CD14 MΦs. JAG1 and Notch3 are overexpressed on all FLS subgroups. And it is primarily enhanced by TLR4 connections. Interestingly, JAG1, DLL1/4, and Notch1/3 exist on the RA VEC, and their expressions are reconfigured with each other by TLR4/5 connections in the VEC (Zack et al., 2024). In addition, Syntenin-1 upregulates the transcription of IRF1/5/7/9, IL-1β, IL-6, and CCL2 via SRC-1 junction and HIF1α or mTOR activation, thereby exacerbating the inflammation of VEC and RA FLS. The VEGFR1/2 and Notch1 networks were found to be responsible for crosstalk between Syntenin-1 reconnected VECs and RA FLS (Meyer et al., 2024). Since current therapies are ineffective against the expression of Syntenin-1 and SDC-1 in RA synovial tissue and blood, targeting this pathway and its interconnected metabolic intermediates may provide a new therapeutic strategy. During the pathogenesis of RA, urokinase-type plasminogen activator (uPA) secreted by neutrophils, chondrocytes and monocytes interacts with uPAR, a receptor expressed on macrophages, FLS, chondrocytes and VECs, thereby secreting a variety of cytokines, chemokines, growth factors and MMPs to promote the progression of RA (Dinesh and Rasool, 2018). Crosstalk between VEC and FLS, macrophages affects the disease course of RA, and targeting this pathway and its interconnected receptors or ligands may provide a novel treatment strategy for RA.

## 4 Crosstalk between VEC and lymphocytes (T cells, B cells, etc*.*)

The interplay between VECs and lymphocytes (T cells, B cells) drives synovial inflammation and angiogenesis in RA (Floudas et al., 2022). T cells activated by cytokines, endothelial transport, extracellular matrix, or autoantigens can interact through cell membranes mediated by β-integrins and membrane cytokines to promote the production of TNF-α and MMPs by cytokines, especially macrophages and FLS (Elshabrawy et al., 2017). This interaction establishes a feedforward loop: VEC-derived cytokines further activate T cells, amplifying synovial inflammation. Similarly, in human synovial fibroblast cell line MH7A transfected with SV40 T antigen, TNF-α increased the expression and transcriptional activity of B cell-derived factors like BAFF (B cell-activating factor) and VEGF. BAFF indirectly modulates VEC function by stimulating synovial fibroblasts to release VEGF, which sustains endothelial activation and vascular permeability (Lee et al., 2013). TNF-α-induced BAFF expression and BAFF-mediated VEGF expression in synovium may crosstalk to maintain the ability of such cells to protect B cells from apoptosis and nutrient and oxygen supply in the inflammatory microenvironment. Despite targeting VEGF, current anti-angiogenic therapies still face limitations. For example, compensating upregulation of alternative angiogenic signals (e.g., BAFF, FGF2) through lymphocyt-VEC crossinuations can reduce drug efficacy. In microenvironmental adaptation, chronic TNF-α exposure induces BAFF/c-Fos signaling in lymphocytes and FLS, maintaining VEGF production even when VEGF is blocked. In addition, resistance arises from overlapping FLS-lymphocyte-VEC interactions, requiring a multi-target approach.

While this section emphasizes VEC-lymphocyte interactions, fibroblast-like synoviocytes (FLS) act as critical intermediaries. RA FLS cultured in the presence of CD40 ligand transfected (CD40L+) L cells increased VEGF production by a factor of 4.1 compared to the composition level of unstimulated FLS. CD40L on T cells upregulates VEGF produced by FLS. The interaction between CD40 on FLS and CD40L expressed on activated T lymphocytes may be directly involved in RA angiogenesis by enhancing VEGF production (Cho et al., 2000). In addition, Activated CD4^+^ T cells and FLS synergistically upregulate adhesion molecules (ICAM-1, VCAM-1) on VECs, facilitating leukocyte recruitment and cytokine release (TNF-α, IFN-γ, IL-17A, IL-6, IL-8) that perpetuate angiogenesis (Petrasca et al., 2020).

## 5 Post-translational modifications interfere with VEC function in RA

**FIGURE 4:**
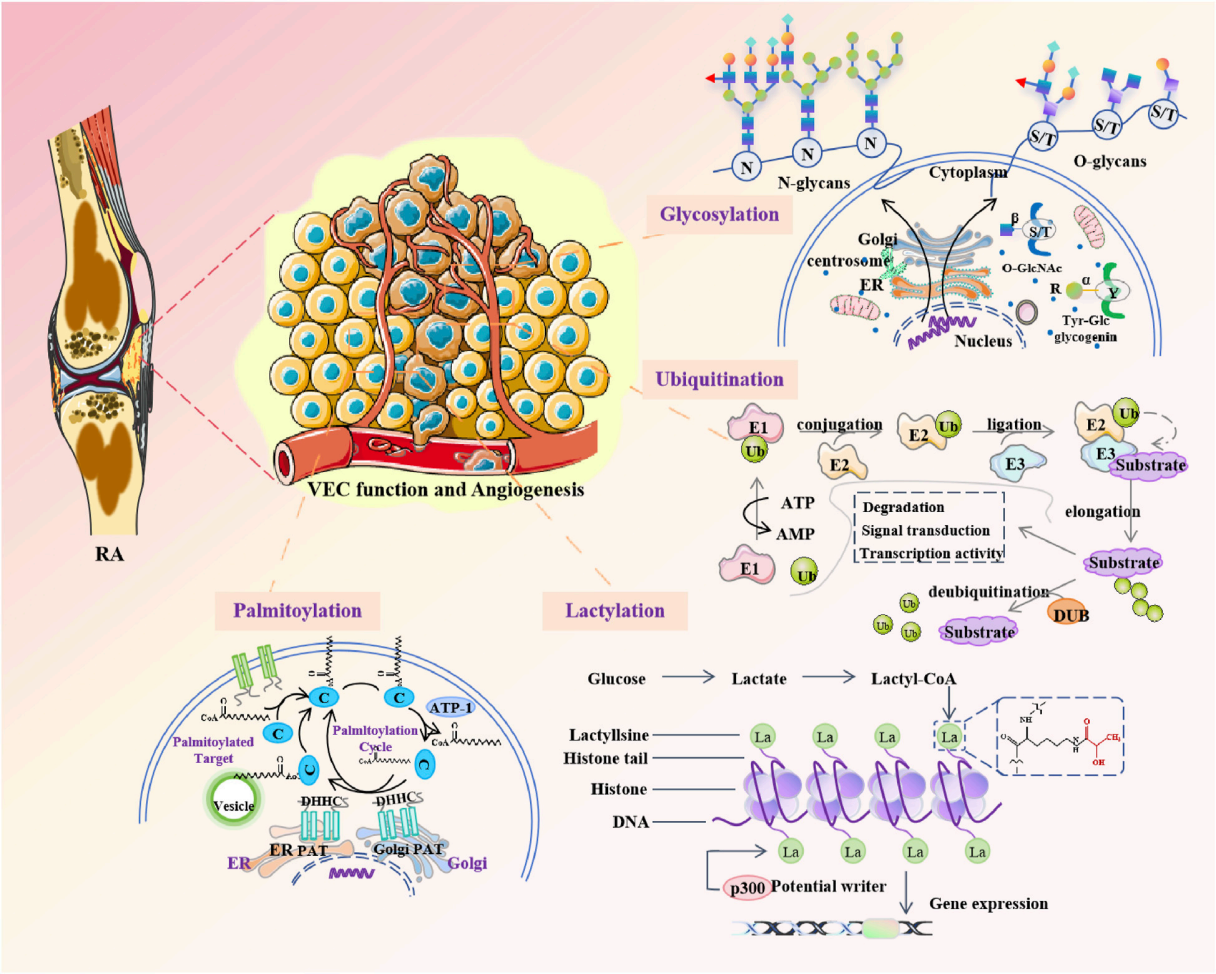
Posttranslational modifications are involved in regulating VEC function and angiogenesis in rheumatoid arthritis.

Post-translational modifications (PTMs) are chemical modifications that play a key role in functional proteomes, further contributing to increased proteomic complexity from the genomic level (Lee et al., 2023). Due to the existence of a large number of different PTMs, it is not possible to conduct a comprehensive review of all possible protein modifications here.This review focuses on PTMs, which are at the forefront of protein research, exploring their role in VEC function and angiogenesis in RA (Figure 4).

### 5.1 Ubiquitination

Ubiquitination fine-tunes endothelial barrier function by modulating Rho GTPases and connexins (Majolée et al., 2019). In RA, overexpression of Rho family GTPase 3 (RND3) reduces proliferation, migration, invasion, and inflammation of RA FLS (Dai et al., 2022). Ubiquitination may affect FLS and VEC function in RA by regulating RND3. The ubiquitin-proteasome system (UPS) plays a central role in fine-tuning the function of core pro-angiogenic proteins, including VEGF, VEGFR-2, angiogenesis signaling proteins (such as the PLCγ1 and PI3 kinase/AKT pathways), and other non-VEGF angiogenesis pathways (Rahimi, 2012). Dysangiogenesis of mesenchymal stem cells (MSCs) is closely associated with inflammation and bone metabolism disorders in patients with various autoimmune diseases. SMAD-specific E3 ubiquitin ligase 2 (Smurf2) overexpression in RA may destabilize PTX3 levels in VECs, exacerbating synovial inflammation and vascular permeability (Ma et al., 2021; Boutet et al., 2021). In RA, these results may provide new insights into whether Smurf2 can similarly influence angiogenesis by acting as an E3 ubiquitin ligase to regulate levels of PTX3 in VEC.

### 5.2 Glycosylation

Targeting metabolism is a new anti-angiogenesis paradigm, particularly by inhibiting energy metabolism and glycosylation, from the perspective of maintaining a delicate balance between the beneficial and harmful effects of excessive angiogenesis in patients (Bousseau et al., 2018). Monocyte chemoattractant protein-1 (MCP-1/CCL2) is a potent monocyte chemoattractant, mainly produced by macrophages and VECs. Patients with RA have significantly higher levels of MCP-1/CCL2 in synovial fluid compared to patients with osteoarthritis or other forms of arthritis. Abnormal glycosylation of MUC3 and MCP-1/CCL2 proteins enhanced monocyte chemotaxis and synovial angiogenesis of RA (Korchynskyi et al., 2023). Mucins(MUC) are a class of highly glycosylated proteins that protect epithelial membranes and serve as ligands for cell adhesion. MUC3 was highly expressed in RA macrophages and fibroblasts compared with normal synovial endomyocytes. The expression level of MUC5AC is low in synovial lining cells, macrophages and endothelial cells of RA, and almost no expression in normal synovial tissue (Volin et al., 2008). Two glycosylated proteins, MUC3 and MUC5AC, play a role in the angiogenesis mechanism of RA, suggesting that novel pharmacological strategies targeting glycosylation can be used to reduce excessive angiogenesis in pathological conditions.

### 5.3 Lactylation

Lactic acid can promote a range of carcinogenic processes, including angiogenesis, invasion, metastasis, and immune escape (Zhang et al., 2019). The rate of lactic acid production is closely related to the rate of cell anabolism and proliferation, but whether the accumulated lactic acid can directly affect the proliferation of VEC has not been reported. Lactylation has been demonstrated to promote tumor progression by hinding the function of T cells and natural killer (NK) cells or supporting the inhibition of tumor-associated macrophages (TAMs), myeloid derived suppressor cells (MDSCs), and regulatory T cells (Tregs) (Fan et al., 2023). Excess lactic acid suppresses the T-cell-mediated immune response (Chen et al., 2022). Lactate inhibits the production of IFN-γ, TNF-α, and IL-2 triggered by T cell receptor (TCR) and impairs the function of cytotoxic T lymphocytes by inhibiting the phosphorylation of the p38 signaling protein. Lactic acid also induces T cell apoptosis by reducing the level of nicotinamide adenine dinucleotide (NAD (+)). In addition, histone lactylation promotes malignant progression by promoting the expression of ubiquitin specific peptidase 39 (USP39) targeting the PI3K/AKT/HIF-1α signaling pathway in endometrial cancer (Wei et al., 2024). The study found that dexamethasone can prevent asthma by regulating the HIF-1α-glycolytic-lactic axis and protein lactylation (Chen et al., 2024). The regulation of the HIF-1α-lactate axis by semasone highlights therapeutic potential, although the RA-specific lactation mechanism remains under-explored.

### 5.4 Palmitoylation

Junctional adhesion molecule C (JAM-C) is an immunoglobulin superfamily protein expressed in epithelial cells, VEC and leukocytes. JAM-C underwent S-palmitoylation on two near-membrane cysteine residues Cys-264 and Cys-265 (Aramsangtienchai et al., 2017). Leukocyte infiltration into RA synovium is a multi-step process in which leukocytes leave the blood and invade the synovium, and leukocyte transendothelial migration and adhesion to RA synovium require adhesion molecules on the surface of VEC and FLS. RA synovial VECs exhibit elevated JAM-C, facilitating myeloid cell adhesion and inflammation. The adhesion of myeloid U937 cells to RA synovial tissue FLS and RA synovial tissue depended on JAM-C (Rabquer et al., 2008). Inhibiting JAM-C palmitoylation could mitigate leukocyte recruitment while preserving physiologic vascular functions. However, whether S-palmitoylation of JAM-C is involved in leukocyte infiltration and angiogenesis in RA deserves further study. Furthermore, PTM/epigenetic drugs often lack cell-type precision, risking off-target effects (e.g., HDAC inhibitors impairing immune tolerance).

## 6 Targeted angiogenesis therapy for RA

**FIGURE 5:**
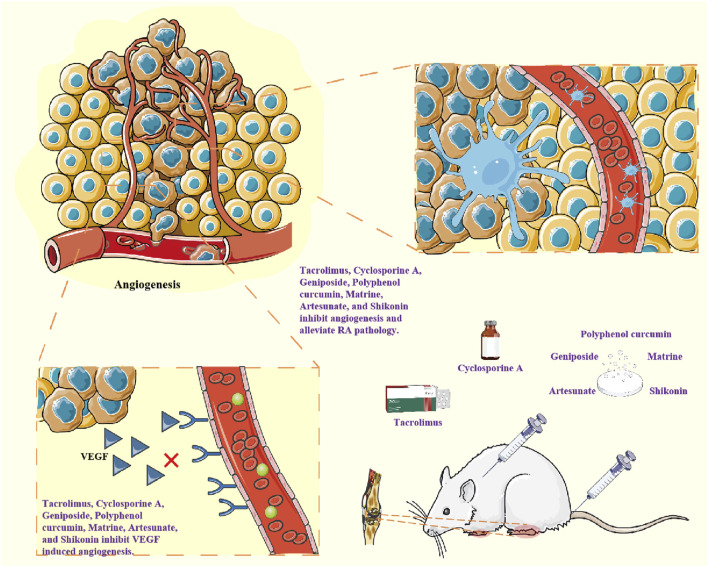
Drugs inhibit the expression of pro-angiogenic factors such as VEGF and inhibit angiogenesis, thereby exerting anti-RA effects.

Standard treatment for RA includes methotrexate and several disease-modifying anti-rheumatic drugs that target key inflammatory molecules that promote RA. These drugs have significant therapeutic effects, but are often associated with significant side effects, and these biologics do not bring sustained relief after discontinuing (Abbasi et al., 2019). VEC-mediated angiogenesis plays a key role in the pathogenesis of RA, and the crosstalk between VEC and FLS and other cells affects the RA process, so targeting angiogenesis related cells and cytokines or pathways may be an important clinical therapeutic target for RA (Figure 5; Table 1).

**TABLE 1:** Drugs used to treat rheumatoid arthritis by antiangiogenesis.

Drug Category	Agent	Mechanism/Target	Key effects
Conventional DMARDs	Tacrolimus(FK506)	Inhibits JNK/p38 MAPK	Reduces ANG-1, Tie-2, VEGF (Choe et al., 2012)
	Cyclosporin A	Suppresses AP-1/VEGF via cAMP	Blocks calcineurin-dependent angiogenesis (Cho et al., 2002)
Biologics	Tocilizumab	Anti-IL-6R antibody	↓ EMMPRIN/CD147; ↑ miR-146a-5p/miR-150-5p (Zisman et al., 2021)
Natural Compounds	Chinese monkshood Decoction	PI3K/AKT/mTOR/HIF-1α pathway	Reduces ANG-1, VEGF (Ba et al., 2021)
	Geniposide	Upregulates PTEN; inhibits PI3K/Akt	Restores pro-/anti-angiogenic balance (Bu et al., 2022; Wang et al., 2021a)
	Polyphenol curcumin	Inhibits NF-κB activation and induces ERK1/2 dephosphorylation	Eliminates the effect of IL-18 on VEGF production (Kloesch et al., 2013; Cho et al., 2006)
	Matrine	Targets HIF-VEGF-Ang axis	↓ IL-1β, VEGF, HIF-α in CIA rats (Ao et al., 2022)
	Shikonin	Downregulates PI3K and Akt while up-regulating PTEN	inhibits TNF-α-induced HUVEC cell migration, invasion, adhesion, and tube formation (Liu et al., 2020)
Repurposed Drugs	Artesunate	Inhibits PI3K/Akt/HIF-1α	↓ VEGF/IL-8 in hypoxic RA-FLS (He et al., 2011)
	Arsenic trioxide (As2O3)	Suppresses TSP-1, TGF-β1, VEGF	Blocks RA-FLS/HDMEC crosstalk (Zhang et al., 2017)
Metabolic Modulators	Dimethyl Malonate	Inhibits succinate/HIF-1α	Disrupts energy metabolism-driven angiogenesis (Li et al., 2018)
	Resveratrol	Activates SIRT1	Suppresses glycolysis in RA-VECs (Jiang et al., 2023b)

### 6.1 Conventional and biologic therapies

Tacrolimus(FK506) is a calcineurin inhibitor. Tacrolimus(FK506) inhibits IL-1β-induced ANG1, Tie-2 receptors and VEGF by blocking phosphorylation of JNK and p38, but not ERK phosphorylation (Choe et al., 2012). The inhibitory effect of cyclosporin A on VEGF synthesis depends on calcineurin. Inhibition of cyclosporin A is associated with reduced binding activity of AP-1 to VEGF promoter in a CAMP-dependent manner. cyclosporin A exerts anti-angiogenic effects by inhibiting AP-1 mediated VEGF expression in RA FLS (Cho et al., 2002). Tocilizumab is an anti-IL-6 receptor antibody. Tocilizumab treatment decreased circulating EMMPRIN/CD147 levels in serum samples from RA patients, enhanced the expression of circulating miR-146a-5p and miR-150-5p, and reduced angiogenic potential, which was manifested by a reduced number of tubular structures formed by the EaHy926 endothelial cell line (Zisman et al., 2021).

### 6.2 Natural and herbal agents

Traditional Chinese medicine and natural medicine have low toxicity in treating RA (Huang et al., 2023). Chinese monkshood Decoction alleviated RA by regulating PI3K/AKT/mTOR/HIF-1α pathway, inhibiting the expression of VEGF, ANGI and other angiogenic factors treating the decoction and inhibiting angiogenesis in MH7A cells (Ba et al., 2021). Geniposide improved the degree of arthritis and angiogenesis in AA rats, inhibited the proliferation and migration of HUVEC and the angiogenesis, and played an anti-angiogenic effect *in vitro*. Geniposide upregulated the expression of PTEN and inhibited the activation of PI3K-Akt signal, thereby inhibiting RA angiogenesis *in vivo* and *in vitro* (Bu et al., 2022). At the cellular level, TNF-γ enhanced VEGF/SphK1/S1P pathway activation in FLS and VEC co-culture models *in vitro*. Geniposide could induce VEGF downregulation in FLS, restore the dynamic balance of pro-/anti-angiogenic factors, inhibit SphK1/S1P signal in VEC, and lead to the decrease of proliferation activity, migration ability, tubeforming ability and S1P secretion ability of VEC cells (Wang et al., 2021b). Polyphenol curcumin has significant anticancer, anti-inflammatory and pro-apoptotic properties. Curcumin can effectively block the expression of IL-6 induced by IL-1β and myristate (PMA) in MH7A cells and RA-FLS. Curcumin inhibits NF-κB activation and induces ERK1/2 dephosphorylation (Kloesch et al., 2013). In addition, curcumin, as a specific inhibitor of transcription-activating protein 1(AP-1), dose-dependently eliminates the effect of IL-18 on VEGF production. The dose-dependent increase of IL-18 in VEGF production was associated with increased binding activity of AP-1 to VEGF promoter sites (Cho et al., 2006). Matrine exerts an anti-angiogenic effect by regulating HIF-VEGF-Ang axis and inhibiting PI3K/Akt signaling pathway, inhibits proliferation and migration of RA-FLS and proliferation and lumen formation of HUVEC,and improving RA symptoms (Ao et al., 2022). Shikonin significantly reduced immature blood vessels in the synovial tissue of inflamed joints in CIA rats. Shikonin inhibits TNF-α-induced HUVEC cell migration, invasion, adhesion, and tube formation. Shikonin downregulates PI3K and Akt while up-regulating PTEN in synovial tissue and/or TNF-α-induced HUVEC cells. It also inhibits the phosphorylation and gene levels of TNF-α-induced signaling molecules. Shikonin has anti-angiogenic effects in RA *in vivo*, *in vitro* and *in vitro* by blocking PI3K/AKT and MAPKs signaling pathways (Liu et al., 2020).

### 6.3 Repurposed drugs

The antimalarial drug artemisinin and its derivatives have anti-angiogenic effects. Artesunate reduces VEGF and IL-8 secretion and HIF-1α translocation in TNF-α or hypoxic-stimulated RA FLS in a dose-dependent manner. Artesunate prevents Akt phosphorylation. PI3 kinase inhibitor LY294002 inhibits VEGF and IL-8 secretion and HIF-1α expression induced by TNFα or hypoxia, suggesting that inhibiting PI3 kinase/Akt activation may inhibit VEGF and IL-8 secretion and HIF-1α expression induced by TNFα or hypoxia (He et al., 2011). Arsenic trioxide (As2O3) is an attractive drug for the treatment of some cancers. TSP-1, TGF-β1, CTGF and VEGF were increased in the supernatant of RA-FLS and human skin microvascular endothelial cells (HDMEC) co-culture. Co-cultured RA-FLS and HDMEC supernatants induced increased migration, tube formation and microvascular germination of HDMEC. As2O3 has a significant anti-angiogenic effect on the synovium of RA-FLS and CIA mice by inhibiting the RA angiogenesis function modules of TSP-1, TGF-β1, CTGF and VEGF, and may have the potential to treat RA other than cancer treatment (Zhang et al., 2017).

### 6.4 Metabolic and novel pathway inhibitors

Succinic acid can act as a signaling molecule that links metabolic reprogramming to angiogenesis. Intracellular succinic acid promotes VEGF production and induces HIF-1α-induced angiogenesis in VEC, while extracellular succinate activates the succinic acid receptor G protein-coupled receptor 91 and induces VEGF production, jointly disrupting energy metabolism and exacerbating inflammation and angiogenesis in RA synovium. Succinate dehydrogenase inhibitors dimethyl malonate can prevent succinic acid accumulation and inhibit angiogenesis by blocking the HIF-1α/VEGF axis (Li et al., 2018). Metabolomics studies revealed accelerated glycolysis of RA sero-treated HUVEC, leading to ATP accumulation but not affecting GTP levels, a process that can be inhibited by activation of SIRT1. Resveratrol can induce SIRT1 activation to inhibit glycolytic-promoted angiogenesis in RA independent of HIF-1α (Jiang TT. et al., 2023).

## 7 Conclusion and perspectives

VECs are central to maintaining vascular homeostasis, immune regulation and tissue repair. Advances in single-cell RNA sequencing (scRNA-seq) and lineage tracing have revealed VEC heterogeneity, identified disease-associated endothelial subpopulations, and clarified their role as mediators of innate immune cell and synovial microenvironment imbalances in RA (Zhao et al., 2024).

The targeted therapy of RA has not made a good breakthrough at present. Therefore, through in-depth research on the important role of VEC on the imbalance of RA synovial microenvironment, especially the role of various cells and factors in mediating the angiogenesis pathway, Exploring the relationship with other pathways and studying the interaction between VEC, FLS and other cells will provide new ideas and directions for the pathogenesis and treatment of RA.

In addition to PTMs, epigenetic modifications have important effects on VEC behavior in RA. Hypomethylation of pro-angiogenesis genes (e.g., VEGFA, ANGPT2) in RA VEC amplifies synovial angiogenesis. HDAC inhibitors (such as Entesteat) reduce VEGF secretion by restoring acetylation-dependent inhibition at inflammatory sites. As previously mentioned, miRNAs and lncRNAs also affect VEC behavior, and the exosome miR-200a-3p from RA-FLS silences KLF6 in vec and enhances VEGF-driven angiogenesis. LncRNA HOTAIR promotes PI3K/AKT activation through miR-126-3p sponge.

These breakthroughs catalyzed the discovery of novel targets such as PTMs and epigenetic regulators that drive angiogenesis and inflammation in RA. However, translating these insights into effective treatments remains challenging.

In recent years, a large number of anti-angiogenesis drugs targeting angiogenesis related proteins and cytokines have appeared, but limited efficacy and drug resistance are still prominent problems (Iragavarapu-Charyulu et al., 2020; Wu et al., 2020). While anti-angiogenic drugs targeting VEGF, PI3K/AKT, and other pathways have shown promise, their clinical utility in RA is limited by (1) Drug resistance: Complex signaling pathways (e.g., compensatory HIF-1α upregulation during VEGF inhibition): enable escape mechanisms (Musleh Ud Din et al., 2024; Lu et al., 2024); (2) Systemic toxicity: Biologics such as bevacizumab (anti-VEGF) often cause hypertension, impaired wound healing, and immunosuppression, and complicate long-term use (Syrigos et al., 2011); (3) Lack of specificity: Current treatments extensively inhibit angiogenesis, disrupt physiologic vascular repair and exacerbate synovial hypoxia; (4) Limited persistence: Transient efficacy requires repeated dosing, increasing the risk of toxicity without addressing the root cause of VEC dysfunction.

To overcome the current barriers to treatment, a multi-pronged strategy is essential. Precise targeting can be used to identify RA-specific VEC subsets using scRNA-seq, enabling spatially-resolved therapies such as nanoparticle-delivered siRNA against pro-angiogenic lncRNA HOTAIR or lactation inhibitors. Dual pathway inhibition - for example, co-targeting VEGF and IL-6/IL-17 signaling pathways - may reduce drug resistance; tocilizumab in combination with PI3K inhibitors disrupts cytokine-driven inflammation and metabolic reprogramming. PTMs based therapies, such as Smurf2 inhibitors that modulate ubiquitination or dexamethasone analogues that target pathologic lactation, offer pathways to restore VEC homeostasis with fewer off-target effects. In addition, given the female predominance of RA, sex-specific approaches are critical: Exploring estrogen regulation of VEC glycosylation and miRNA networks could provide ideas for gender-tailored treatments.

Furthermore, insights from RA VEC biology hold promise in multiple areas. Angiogenesis plays an important role in tumor growth and spread, and how to inhibit tumor angiogenesis to block tumor blood supply and thus inhibit its growth may be a promising treatment (Cai et al., 2024). Cardiovascular diseases such as heart disease and coronary heart disease are related to vascular dysfunction. Studying the mechanism of angiogenesis can help develop new therapeutic strategies and improve the prevention and treatment of cardiovascular diseases. Exploring how to promote angiogenesis within artificial tissues and organs to provide nutrients and oxygen to promote their function and growth is of key significance for tissue engineering and organ regeneration. Several inhibitors of angiogenesis have been developed and are used to treat certain cancers and other diseases. However, although there have been studies on drug therapy mechanisms targeting VEC and angiogenesis pathway in the treatment of RA, there is still no substantive breakthrough. It is exciting that the PTMs related to the function of VEC may contribute to further research on the pathogenesis of RA and explore new diagnostic and therapeutic targets for RA.
